# Refractory Inappropriate Sinus Tachycardia Treated with Pulsed-field Ablation of the Sinus Node: A Breath of Fresh Air

**DOI:** 10.19102/icrm.2024.15032

**Published:** 2024-03-15

**Authors:** Robert N. Kerley, Noel Fitzpatrick, Joseph Galvin

**Affiliations:** 1 Mater Misericordiae University Hospital, Dublin, Ireland

**Keywords:** Catheter ablation, inappropriate sinus tachycardia, sinus node modification

## Abstract

The pathogenesis of inappropriate sinus tachycardia is not well understood, and the symptoms of inappropriate sinus tachycardia can be difficult to manage. Here, we present a case of inappropriate sinus tachycardia refractory to medical therapy and discuss our approach to sinus node modification by catheter ablation.

## Introduction

Inappropriate sinus tachycardia (IST) is a clinical syndrome for which the pathogenesis is not well understood. It is a diagnosis of exclusion defined by otherwise unexplained persistent or paroxysmal sinus tachycardia with a resting heart rate of >100 bpm.^1^ The symptoms associated with IST can be distressing and difficult to manage. The cause of IST is considered multifactorial, with autonomic dysfunction being the central abnormality. Management can be challenging, with many cases being refractory to medical management.^2^ Catheter-based ablation of the sinus node has limited efficacy for the treatment of IST, often requiring repeated procedures.^1–3^ Here, we present a case of IST refractory to medical therapy and our approach to sinus node modification by catheter ablation.

## Case presentation

We present a case of a 27-year-old woman with a history of IST. She had a background medical history of Ehlers–Danlos type 3 but no history of structural heart disease.

Her symptoms were debilitating shortness of breath with climbing hills or walking on the flat for >10 min. Her condition was unresponsive to oral β-blocker and ivabradine therapy, but she did respond to intravenous β-blocker therapy, with a significant improvement in her exercise tolerance, suggesting that her symptoms were entirely related to her IST. Unresponsive to medical therapy, she had an electrophysiology (EP) study, which ruled out other causes of supraventricular arrhythmia and demonstrated a right atrial superior to inferior activation sequence with attempted radiofrequency ablation (RFA) of the sinus node, which was ineffective. Activation mapping before and after RFA ablation showed that her sinus node had migrated caudally along the crista terminalis and was now situated close to the phrenic nerve, rendering the ablation procedure an unfavorable option. Resistant to RFA sinus node modification, she was referred for cardiothoracic surgery and underwent right-sided sympathectomy due to ongoing refractory, debilitating symptoms. Unfortunately, however, she continued to have a mean resting heart rate of 125 bpm and recorded rates of 150–180 bpm while walking in the office.

**Figure 1:**
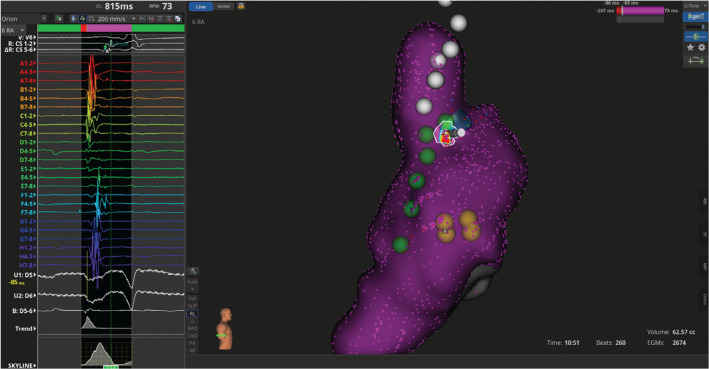
Pre-ablation activation map with signals showing earliest site in the sinoatrial node.

**Figure 2:**
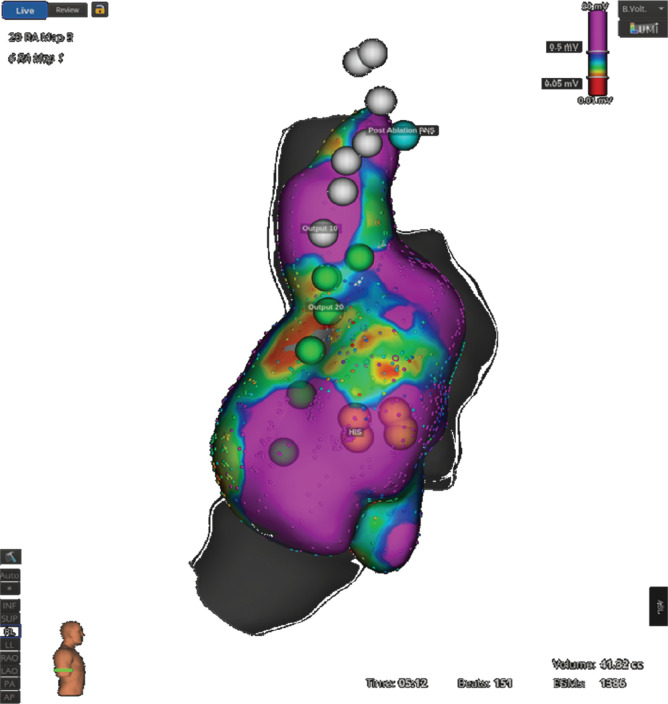
Voltage of the right atrium showing areas of low voltage, phrenic nerve pacing sites, and the His cloud site.

**Figure 3:**
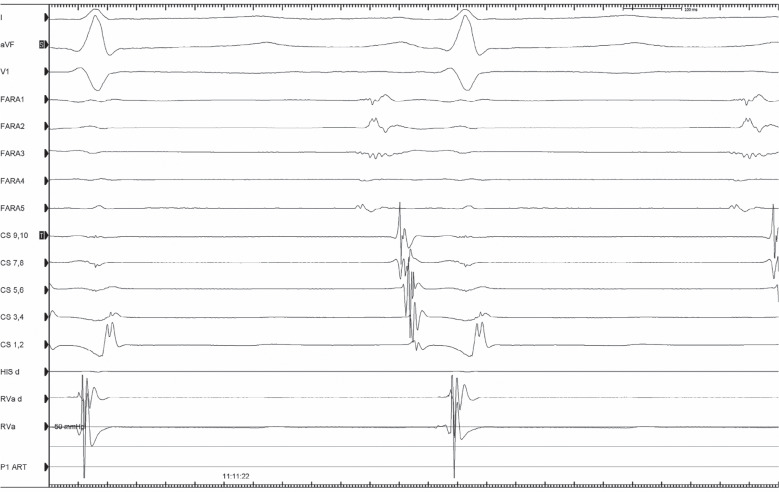
Fractionated electrogram showing a 40-ms pre-surface P-wave representing local endocardial activation in the upper crista terminalis and targets for ablation.

**Figure 4:**
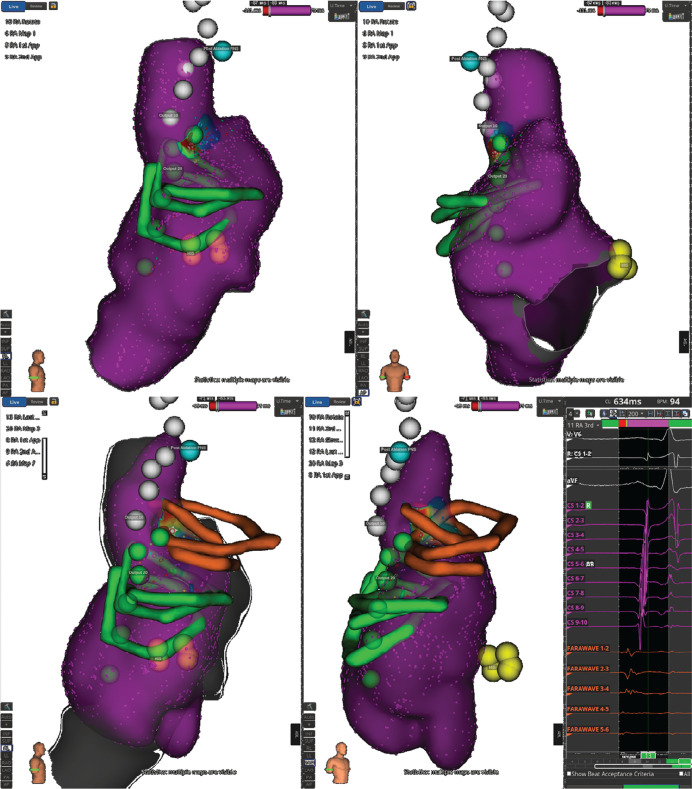
Activation maps showing target sites for pulsed-field ablation using the FARAPULSE™ system (Boston Scientific, Marlborough, MA, USA).

**Figure 5:**
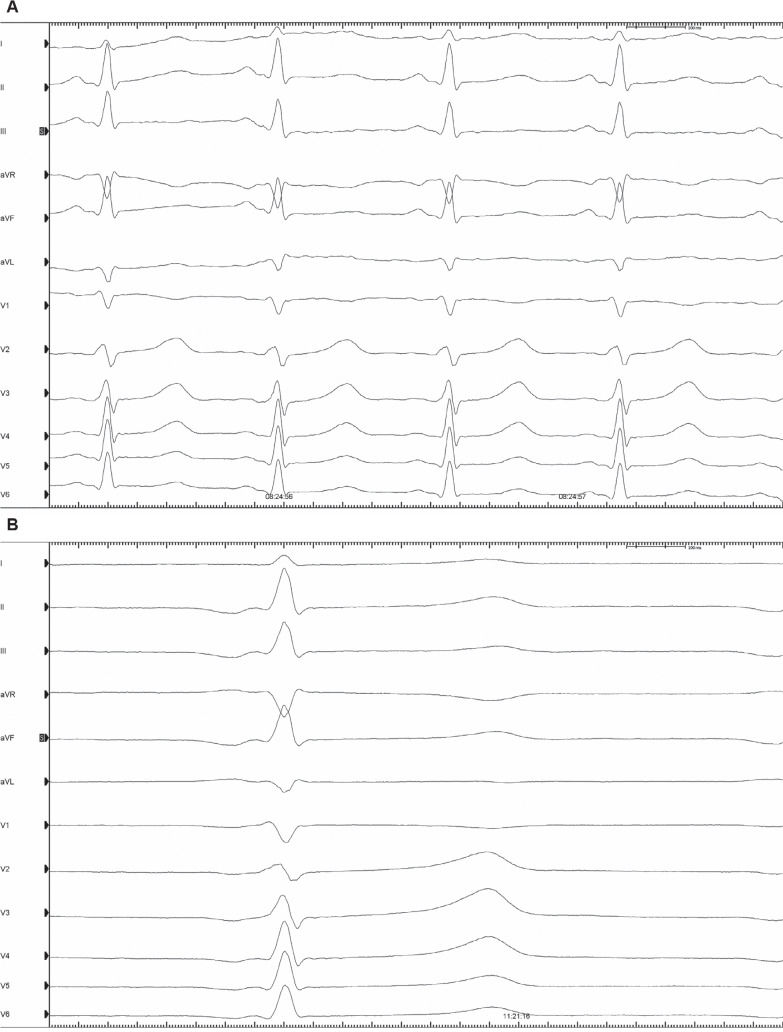
Twelve-lead electrocardiograms recorded **(A)** pre-ablation and **(B)** post-ablation, with ablation resulting in a low right atrial rhythm.

Following a long discussion, which included alterations to medications, we put forward an option to perform the procedure using an alternative strategy involving pulsed-field ablation (PFA), as this approach would theoretically not carry the same risk to the phrenic nerve as RFA. The patient agreed and she was brought to the EP laboratory, where the site of earliest activation was identified in the superior crista terminalis and the course of the phrenic nerve was identified using high-output pacing **(Figures 1 and 2)**. Sites where local endocardial activation preceded the P-wave by 15–60 ms were targeted for ablation with PFA **(Figure 3)**. PFA of the sinus node, superior crista terminalis, and ostial superior vena cava resulted in a low right atrial rhythm, with her sinus rate decreasing from 116 bpm at baseline to 86 bpm post-PFA **(Figures 4 and 5)**. As such, the patient had a successful PFA modification of the sinus node with a significant improvement in her symptoms. After this procedure, her baseline heart rate was much improved, but she continued to have issues with exertional sinus tachycardia and underwent further PFA modification of the sinus node with complete resolution of her symptoms. The procedure was well tolerated on both occasions, with no peri-procedural complications.

## Discussion

As far as the authors are aware, this is the first published case of IST treated with PFA of the sinus node. Endocardial RFA modification of the sinus node has limited success rates with significant complication rates.^3,4^ The advantage of the PFA approach is that it will cause local tissue issue injury via electroporation but will not affect any collateral structures, which, in this case of sinus node modification, includes the phrenic nerve. Given the propensity for the creation of transmural scars with PFA, we anticipated the need for permanent pacemaker insertion to provide back-up atrial pacing; however, this was fortunately not required. Furthermore, our patient tolerated a repeat procedure with complete resolution of symptoms, suggesting that PFA modification of the sinus node is a safe and effective treatment for IST.
